# The impact of removal of the seasonality formula on the eligibility of Irish herds to supply raw milk for processing of dairy products

**DOI:** 10.1186/s13620-017-0083-z

**Published:** 2017-02-23

**Authors:** Caroline Fenlon, Luke O’Grady, Finola McCoy, Erik Houtsma, Simon J. More

**Affiliations:** 1 UCD School of Computer Science, University College Dublin, Dublin, Ireland; 2 UCD School of Veterinary Medicine, University College Dublin, Dublin, Ireland; 3 Animal Health Ireland, 4-5 The Archways, Carrick-on-Shannon, Co. Leitrim Ireland; 4 Centre for Veterinary Epidemiology and Risk Analysis, UCD School of Veterinary Medicine, University College Dublin, Dublin, Ireland

**Keywords:** Somatic cell count, Milk quality, Legislation, Compliance, Ireland, Seasonality formula

## Abstract

**Background:**

The dairy industry in Ireland is expanding rapidly, with a focus on the production of high quality milk. Somatic cell counts (SCC) are an important indicator both of udder health and milk quality. Milk sold by Irish farmers for manufacture must comply with EU regulations. Irish SCC data is also subject to a monthly seasonal adjustment, for four months from November to February, on account of the seasonality of milk production in Ireland. In a recent study, however, there was no evidence of a dilution effect on SCC with increasing milk yield in Irish dairy cattle. The aim of this paper is to estimate the impact of removal of the seasonality formula on the eligibility of Irish herds to supply raw milk for processing of dairy products.

**Methods:**

Bulk tank SCC data from 2013 were collected from 14 cooperatives in Ireland. The geometric mean of SCC test results was calculated for each calendar month. We then calculated the number of herds and volume of milk supplied falling in three SCC categories (<200,000, 200,000–400,000, >400,000 cells/mL) in Ireland during 2013 based on their geometric mean SCC every month. Each herd was assigned an ‘eligibility to supply’ status (always compliant, under warning (first warning, second warning, third warning) and liable for suspension) each month based on their 3-month rolling geometric mean, using methods as outlined in EU and Irish legislation. Two methods were used to calculate the 3-month rolling geometric mean. We then determined the number of herds and volume of milk supplied by ‘eligibility to supply’ status in Ireland during 2013. All calculations were conducted with and without the seasonality adjustment.

**Results:**

The analyses were performed on 2,124,864 records, including 1,571,363 SCC test results from 16,740 herds. With the seasonality adjustment in place, 860 (5.1%) or 854 (5.1%) of herds should have been liable for suspension during 2013 if calculation method 1 or 2, respectively, had been used. If the seasonality adjustment were removed, it is estimated that the number of herds liable for suspension would increase from 860 to 974 (13.2% increase) using calculation method 1, or from 854 to 964 (12.9% increase) using calculation method 2.

**Conclusions:**

The modelled impact of such removal would be relatively minor, based on available data, regardless of the method used to calculate the 3-month rolling geometric mean. The focus of the current study was quite narrow, effectively from July to December 2013. Therefore, the results are an underestimate of the total number of herds liable for suspension during 2013. They may also underestimate the true percentage change in herds liable for suspension, with the removal of the seasonality formula. A national herd identifier was lacking from a sizeable percentage of the 2013 bulk tank SCC data, but will be needed if these data are to be meaningfully used for this or other purposes.

**Electronic supplementary material:**

The online version of this article (doi:10.1186/s13620-017-0083-z) contains supplementary material, which is available to authorized users.

## Background

The dairy industry in Ireland is expanding rapidly, with ambitious national targets as outlined in Food Harvest 2020 [1, 2] and Food Wise 2025 [3]. These targets include a 50% increase in milk production by 2020 (based on production in the years 2007–2009). By 2014, the industry had seen a 10% increase in milk volume production [2]. Ireland supplies in excess of 11% of the infant formula traded internationally, and high quality value-added specialist dairy ingredients are also sold into the global beverage, nutritional and bakery sectors. In 2014, the value of exported dairy products and ingredients was estimated to be in excess of €3 billion, a 55% increase since 2009 [4]. Securing a consistent supply of high quality milk is essential for milk processors, who must respond to customer and consumer demand if they wish to capitalise on market opportunities.

Somatic cell counts (SCC), due to inflammation of the udder, are an important indicator both of udder health and milk quality. The total cost of mastitis includes both the direct or failure costs associated with the disease (that is, production losses, culling, and treatment) and the time and money that farmers invest in controlling mastitis (the preventive costs) [5]. The failure costs have been well documented [e.g. 6, 7], including a reduction in yield and an increase in treatment and culling costs. In recent work, Geary et al. have estimated the impact of mastitis (clinical and subclinical) on the net profit of Irish dairy farms [7]. In addition to the quantifiable financial benefits associated with lower SCC herds, there are also advantages such as easier herd management and milking procedures, along with less complex on-farm decision-making and the mental stress associated with this. Geary et al. also showed that using milk of a lower SCC for manufacturing dairy products delivers increased net revenue to the processor, as a result of an associated change in raw milk composition and cheese processing and composition [8, 9].

In addition to customer specification, milk sold by Irish farmers for manufacture must also comply with EU regulations. Regulations 853/2004 and 854/2004 govern the eligibility to supply raw milk for processing, using a three-month rolling average bulk tank SCC as one of the determining criteria. Regulation 854/2004 provides for a further three months to correct the situation in the event of the rolling geometric average at first exceeding the requirements of Regulation 853/2004. Irish SCC data is also subject to a monthly seasonal adjustment, for four months from November to February, as allowed under Commission Decision 96/360/EC. Milk production is highly seasonal in Ireland, and the adjustment was introduced to address the potential effect of dilution on SCC. It has been suggested that SCC is reduced in all cows due to the dilution effect of increased milk yields [10], which is relevant to the milk yield increase observed in Ireland during summer. In winter, therefore, SCC is expected to concentrate (increase) with reducing milk volumes, in a seasonal production system. Until recently, however, there was little supporting research, and none from Ireland. In 2012, Hand et al. suggested that ‘to date, the dilution effect has not been quantified’ [11]. To directly address this knowledge gap, research was recently completed in Ireland, with Boland et al. finding no evidence of a dilution effect on SCC with increasing milk yield in Irish dairy cattle [12]. In other words, there does not appear to be robust scientific support for Commission Decision 96/360/EC.

The aim of this paper is to estimate the impact of removal of the seasonality formula on the eligibility of Irish herds to supply raw milk for processing of dairy products. We also sought to estimate the influence of two different methods to calculate herd elibility to supply. These methods each conform with the primary EU legislation.

## Methods

### The data

The data for this study were collected by the Department of Agriculture, Food and Marine (DAFM) during spring 2014. In total, 14 cooperatives that purchase and/or process raw milk were asked by DAFM to submit the bulk tank data to a standard specification for every milk collection from farms in 2013. This vat or bulk tank-level data included a unique supplier identifier, the date of collection from farm, the volume of milk collected and, when tested, the SCC result. This dataset accounted for 93% of the national milk pool. The dataset received from each cooperative was uploaded securely to the Irish Cattle Breeding Federation (ICBF) database, where all data was combined into a single dataset for cleaning and further analysis.

The raw data consisted of 2,134,474 records of milk volume and SCC tests from 16,740 herds, including missing, invalid and duplicated values for both volume and SCC. The cleaning and reduction of the records consisted of the following steps:

- 59,584 SCC test results recorded as 0 were changed to be missing values.
- 100 negative milk volume records were removed. For 38,108 days with multiple recorded unique volumes from the same herd, the values were added together. If multiple SCC tests were also present, a weighted mean was calculated for the overall milk volume collected that day. In this way, no more than one record with volume and/or SCC was recorded per herd per day.

After cleaning, each observation in the dataset (*n* = 2,124,864) represented a collection day record, consisting of a unique herd number, the date of collection, the total milk volume (litres) collected on that day and, if tested, a somatic cell count record for the collection.

### Data analyses

All calculations were carried out at herd level.

### Preliminary analysis

For a sizeable percentage of the 2013 bulk tank SCC data (57.7% of herds, 49.6% of all herd-month pairs), the national herd identifier was not available (some cooperatives supplied their own randomly generated herd code, which could change year on year). Although a similar dataset was created containing 2014 data, the 2013 data could only be joined with 2014 data for herds where the national herd identifier was available. Therefore, we first investigated SCC and monthly milk volumes in herds with and without herd ID (so-called usable and non-usable records or data) in the 2013 dataset.

Descriptive analysis included calculating summary statistics for both SCC and monthly volume records. Counts of the number of herds were based on herds with a recorded herd identifier (either national herd number, or co-op generated identifier), as the number of herds with no herd identifier could not be calculated. Median values as well as percentiles 5, 25, 50, 75 and 95 are provided to give an idea of the differences between usable and non-usable data. Then we investigated if it was appropriate to use a subsample of the original bulk tank dataset, based on selecting records for which a herd number was available along with two consecutive years of data, in place of the original full dataset. We compared the distributions of usable and unusable records to verify that both samples come from a similar distribution using a two sample Kolmogorov-Smirnov test in SAS 9.3. Assuming both samples come from the same distribution implies that the subsample of usable records could be used in place of the whole dataset, and is representative of the national figures.

### Main analyses

All subsequent analyses were conducted using the full dataset for 2013, including data from herds with and without the national herd identifier. All data manipulation and analyses were carried out using the R statistical programming language [13] (the pseudocode detailing the two methods for calculating the rolling geometric mean herd SCC and monthly herd compliance is available in Additional file 1: Appendix 1). Graphs were created with the ggplot2 package [14].

#### Monthly geometric mean SCC

The geometric mean is calculated using the product of the values, rather than the sum (as is the case with the more commonly-used arithmetic mean). It is calculated as the *n* ^*th*^ root of the product of the *n* numbers to be averaged. Geometric means are less influenced by small numbers of very high values than arithmetic means are and are always lower than the arithmetic mean of the same values.

The geometric mean of SCC test results was calculated for each calendar month. Seasonal adjustments were applied to the monthly geometric mean during January, February, November and December by multiplying the geometric mean of the affected months by the appropriate factor (see Table 1). We then calculated the number of herds and volume of milk supplied falling in three SCC categories (<200,000, 200,000–400,000, >400,000 cells/mL) in Ireland during 2013 based on their geometric mean SCC every month, with and without the seasonality adjustment.

**Table 1:** Seasonality adjustment factors applied during 2013 and 2014 in Ireland

	Adjustment factor	
	2013	2014
January	0.39	0.39
February	0.74	0.75
November	0.78	0.9
December	0.47	0.53

#### Herd eligibility to supply

##### Calculating the 3-month rolling geometric mean SCC

For this study, two different methods were used to calculate the 3-month rolling geometric mean SCC, as outlined below. Calculation method 1 has been described previously [15], whereas calculation method 2 is currently applied by DAFM. The methods differ solely in the manner in which the 3-month rolling geometric mean is calculated, but can have an impact on herd eligibility to supply.

- For calculation method 1, we used all available SCC test results, regardless of whether a corresponding milk volume collection record was available. With this method, we first calculated a monthly geometric mean using all available SCC test results in the month. Seasonal adjustments were then applied to the monthly geometric mean during January, February, November and December by multiplying the geometric mean of the affected months by the appropriate adjustment factor (see Table 1). Finally, the 3-month rolling geometric mean was calculated as the geometric mean of the monthly geometric mean SCC value of the current and two preceding months.
- For calculation method 2, only collection day records with both an SCC result and milk volume were used. Using this method, the 3-month rolling geometric mean was calculated as the geometric mean of all individual SCC test results during the current and two proceeding months. For the SCC test results collected during January, February, November and December, the seasonal adjustment was applied first, by multiplying all individual SCC test results by the appropriate factor (Table 1).

With both of the calculation methods, the rolling geometric mean requires 3 consecutive months with at least one test result during each; therefore, the 3-month rolling geometric mean was not calculable for the first two months in the dataset due to limitations of the dataset. Similarly, the 3-month rolling geometric mean was incalculable during the first two months following a break in supply of at least one calendar month.

In Table 2, we illustrate the calculation of the arithmetic and geometric mean SCC values for a sample herd during the first three months of the year. The two calculation methods were applied for March, the first month with three months of consecutive SCC results.

**Table 2:** Calculating the arithmetic and geometric mean SCC values (* 1000) for a sample herd during the first three months of the year, immediately following a break-in-supply

Month	SCC test results	Seasonal adjustment factor	Monthly arithmetic mean	Monthly geometric mean	Calculation method 1		Calculation method 2	
					Adjusted geometric mean	Rolling geometric mean	Adjusted SCC	Rolling geometric mean
January	438	0.39	403.50	395.62	154.29	-	170.82	-
	540						210.60	
	413						161.07	
	402						156.78	
	283						110.37	
	345						134.55	
February	513	0.74	470.29	460.49	340.76	-	379.62	-
	328						242.72	
	463						342.62	
	662						489.88	
	408						301.92	
	504						372.96	
	414						306.36	
March	659	1.00	402.29	377.65	377.65	270.79	659.00	278.51
	555						555.00	
	394						394.00	
	407						407.00	
	288						288.00	
	226						226.00	
	287						287.00	

The two methods of rolling mean calculation are applied for March, this being the first month with three months of consecutive SCC results

##### Determining herd eligibility to supply

As outlined in relevant European and Irish legislation, herd eligibility to supply milk for processing of dairy products is determined after considering the 3-month rolling geometric mean in the preceding month(s) [15]. In this study, based on the available dataset, in any particular month each herd was assigned an ‘eligibility to supply’ status, either:

- *Compliant,* if the 3-month rolling geometric mean was below 400,000 cells/mL in the preceding month.
- *Under warning,* either:
  - ○ *First warning* if the 3-month rolling geometric mean in the preceding month exceeded 400,000 cells/mL in a herd that was *compliant* in the month prior to that again,
  - ○ *Second warning* if the 3-month rolling geometric mean in the preceding month exceeded 400,000 cells/mL in a herd that was previously under *first warning* (that is, geometric mean exceeding 400,000 cells/mL for two consecutive months), or
  - ○*Third warning* if the 3-month rolling geometric mean in the preceding month exceeded 400,000 cells/mL in a herd that was previously under *second warning* (that is, geometric mean exceeding 400,000 cells/mL for three consecutive months).
- *Liable for suspension,* if the 3-month rolling geometric mean in the preceding month exceeded 400,000 cells/mL in a herd that was previously under *third warning* (that is, geometric mean exceeding 400,000 cells/mL for four consecutive months). A suspended herd was not eligible to supply milk until the 3-month rolling mean again satisfies compliance regulations.
- *Incalculable,* if the 3-month rolling geometric mean could not be calculated (for example, during the first three months following a break in supply).

Separately using each of the two calculation methods, we determined the number of herds and volume of milk supplied by ‘eligibility to supply’ status (always compliant, under warning (first warning, second warning, third warning) and liable for suspension) in Ireland during 2013, with and without the seasonality adjustment. The latter were calculated as previously, but without first applying the seasonal adjustment either to the monthly geometric mean SCC value (Calculation method 1) or to individual SCC values (Calculation method 2) during January, February, November and December.

## Results

**Fig. 1:**
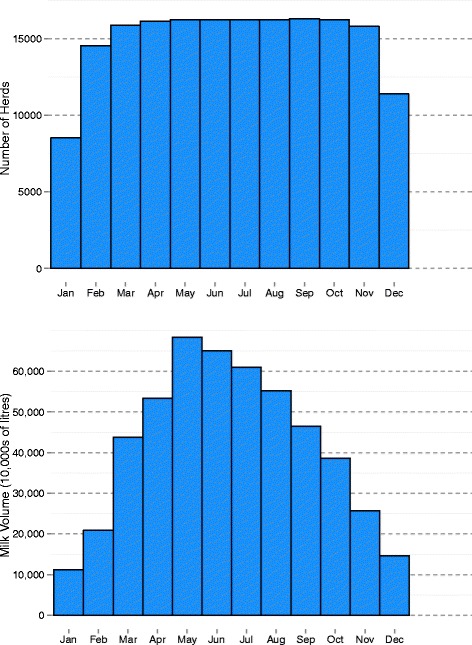
The number of herds supplying (*top*) and total volume of milk supplied (*bottom*) during 2013, by month. 16,740 herds supplied for at least 1 month during 2013

The analyses were performed on 2,124,864 records from 16,740 herds supplying milk to 14 processors. In total, there were 1,571,363 (74.0% of all) records with an SCC test result, 553,501 (26.0% of all) records with volume but no SCC data, and 8947 (0.4% of all) records with SCC but no volume data. The number of herds supplying and the total volume of milk supplied during 2013, by calendar month, is presented in Fig. 1.

### Preliminary analysis

A large number of SCC (58.9%) and monthly volume (58.5%) records was considered to be unusable data. Examination of SCC descriptive statistics showed an arithmetic mean SCC of 214 and 208.4 (x 1000 cells/mL) for usable and unusable records, respectively, but with a high degree of variability. The standard deviation was 123.48 and 123.68, respectively. A clearer difference was shown for the monthly volume data, with arithmetic mean values of 28,231 and 30,778 L for usable and unusable records. Again this came with a high degree of variability. Examination of the median values shows similar results. The median values as well as associated percentiles are presented in Table 3.

**Table 3:** Descriptive statistics of SCC and monthly volume records

Statistic	Usable^a^	Unusable^a^
	6,559 (39.6%) herds	10,020 (60.4%) herds
Somatic Cell Count	Number (%) of herds^b^	
Number of SCC records (%)	71,815 (41.1)	102,810 (58.9)
	SCC (x 1,000 cells/mL)	
Mean (Standard deviation)	214 (123.48)	208.4 (123.68)
5th percentile	70	71
25th percentile	131	129
50th percentile	191.7	186
75th percentile	270	259
95th percentile	427.18	414
Monthly volume (Litres)	Number (%) of herds^b^	
Number of monthly volume records (%)	73,245 (41.5)	103,391 (58.5)
	Litres	
Mean (Standard deviation)	28,230.9 (25,143.56)	30,777.7 (26,903.61)
5th percentile	2,810	2,449
25th percentile	11,080	11,940
50th percentile	22,164	25,131
75th percentile	38,111	42,078
95th percentile	73,243	78,833

^a^Records with or without a national herd identifier were termed usable or unusable data, respectively

^b^The numbers presented are based on records with a herd identifier. The number of herds without a herd identifier cannot be calculated

There was no evidence to suggest that the distribution functions of the subsample dataset and the full bulk tank dataset are the same (Table 4). Thus it was not suitable to use the subsample, with consecutive years of data to resolve the issue of the enforced apparent ‘break in supply’ for all herds immediately prior to January 2013.

**Table 4:** Comparison of distributions of unusable and usable monthly volume and SCC records - annually and monthly

Period	SCC			Monthly volume		
	Number of unusable records^a^	Number of usable records^a^	*p*-value^b^	Number of unusable records^a^	Number of usable records^a^	*p*-value^b^
Annual	102810	71815	<0.001	103391	73245	<0.001
Monthly						
January	4237	4157	<0.001	4366	4169	<0.001
February	8391	5822	<0.001	8445	5838	<0.001
March	9426	6386	<0.001	9462	6400	<0.001
April	9641	6469	0.002	9665	6478	<0.001
May	9684	6307	0.007	9711	6487	<0.001
June	9673	6318	<0.001	9707	6487	<0.001
July	9665	6300	<0.001	9687	6459	<0.001
August	9638	6284	<0.001	9682	6468	<0.001
September	9615	6321	<0.001	9641	6476	<0.001
October	9630	6307	0.005	9662	6469	0.003
November	8845	6222	<0.001	8931	6405	<0.001
December	4365	4922	0.009	4432	5109	<0.001

^a^Records with or without a national herd identifier were termed usable or unusable data, respectively

^b^A *p*-value of <0.05 indicates there is no evidence to suggest the distribution of the usable and all records are the same

### Main analyses

**Fig. 2:**
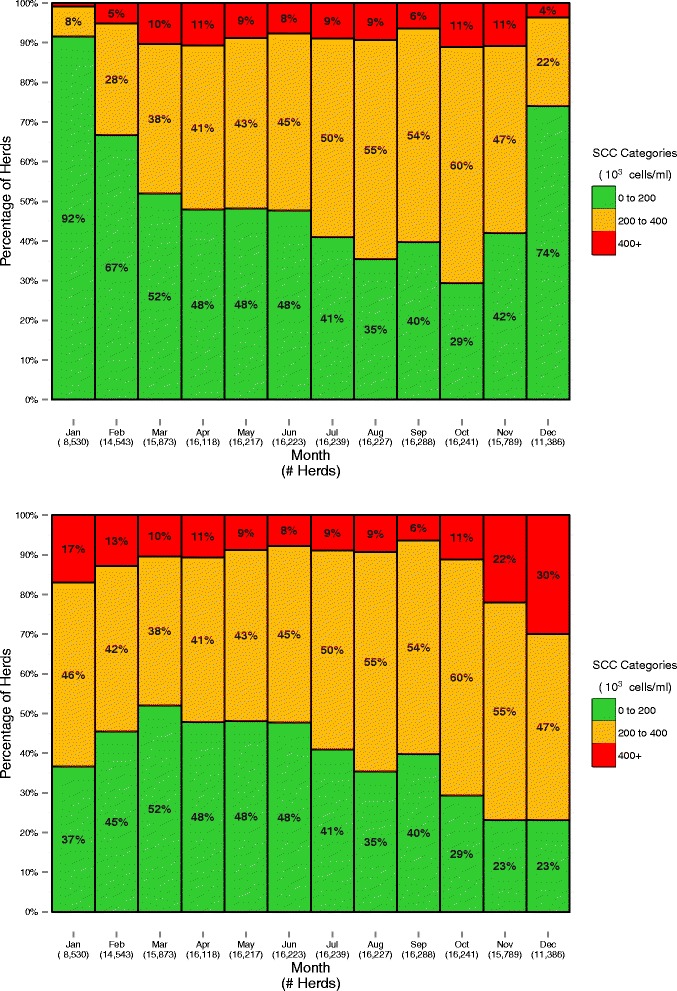
The calculated percentage of Irish herds in different SCC categories during each month of 2013, based on the monthly geometric SCC mean with (top) and without (bottom) application of the seasonal adjustment

**Fig. 3:**
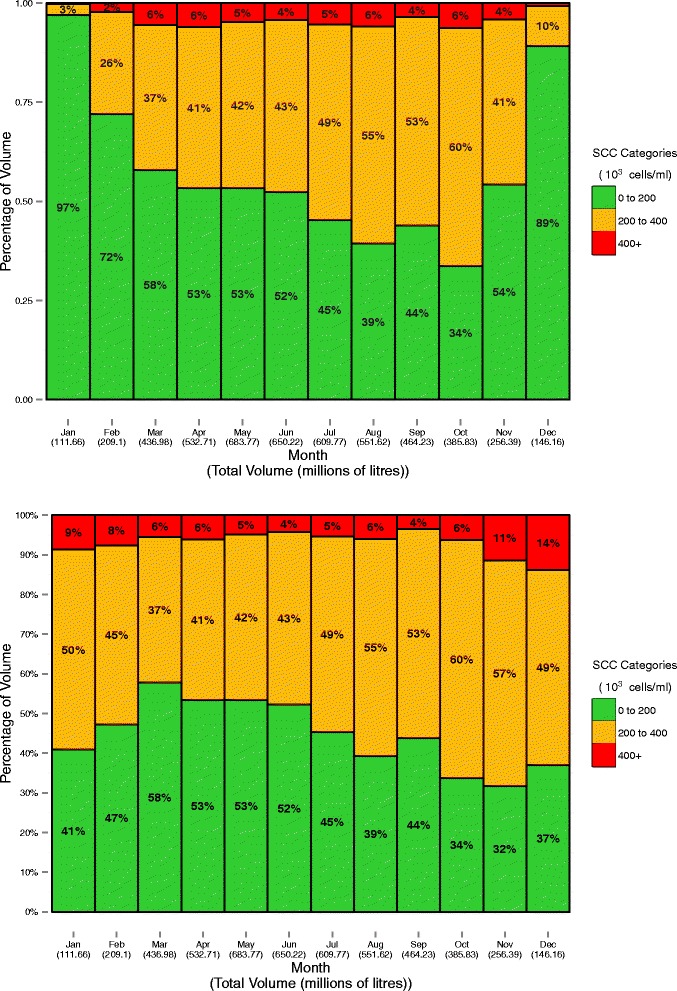
The calculated percentage of milk volume of different SCC categories during each month of 2013, based on the monthly geometric SCC mean with (top) and without (bottom) application of the seasonal adjustment

The proportions of herds and milk volume supplied falling in the three SCC categories described above, based on their geometric mean SCC every month, are shown in Figs. 2 and 3. Geometric means with and without seasonal adjustments applied are presented separately for herds (Fig. 2) and milk volume (Fig. 3).

**Fig. 4:**
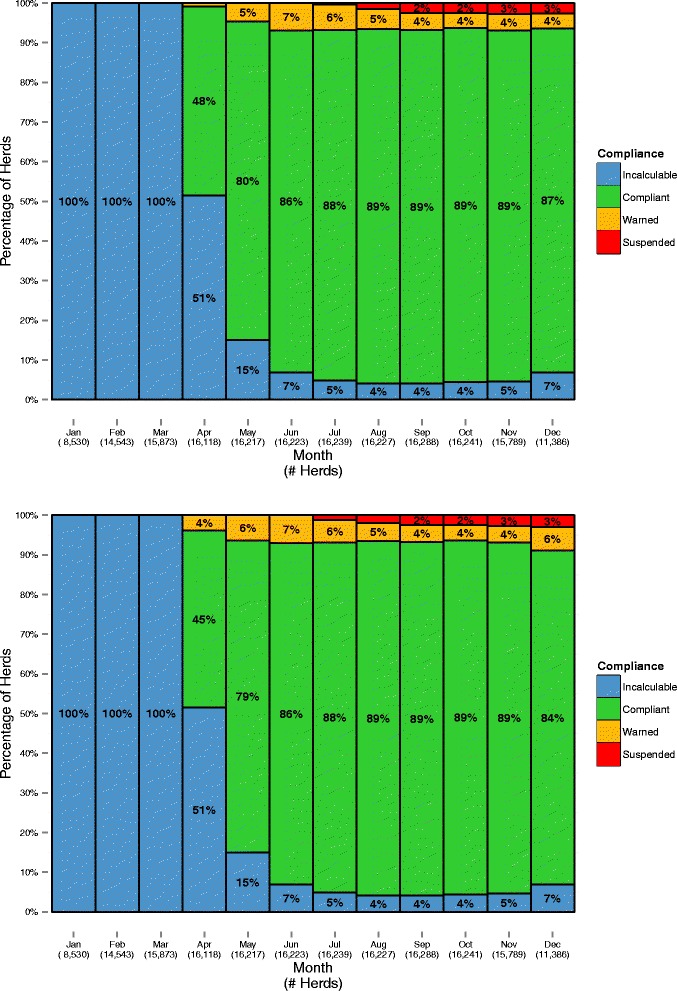
The estimated percentage of Irish herds eligible to supply raw milk for processing of dairy products, by month, using calculation method 2, and with (top) and without (bottom) application of the seasonal adjustment

**Fig. 5:**
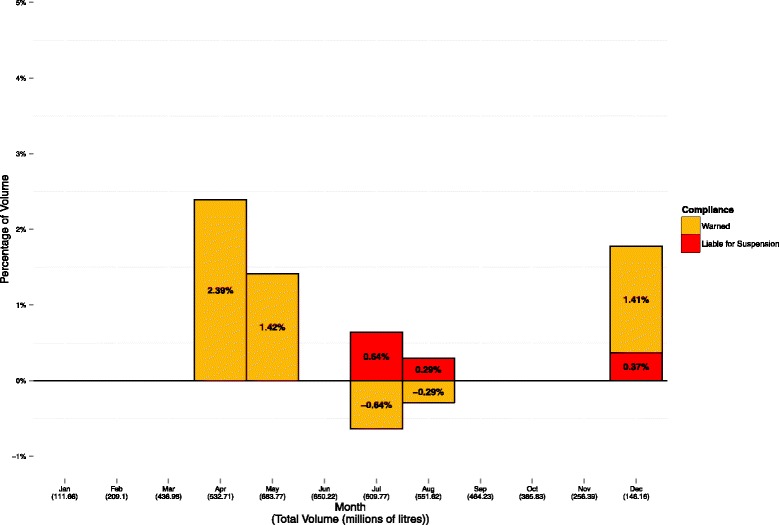
The relative monthly change in the percentage of national volume under warning and liable for suspension following removal of the seasonality adjustment, using calculation method 2

The estimated percentage of herds eligible to supply raw milk for processing of dairy products each month, with and without the seasonality formula applied, are presented in Fig. 4. These were calculated using calculation method 2, however, the results obtained using calculation method 1 (see Additional file 2: Figure S1) were very similar. The relative monthly change in the percentage of the national milk volume under warning and liable for suspension following removal of the seasonality formula, using calculation method 2, is presented in Fig. 5. Similar results were obtained using calculation method 1 (see Additional file 3: Figure S2).

Table 5 presents the estimated number of Irish herds eligible to supply milk for processing of dairy products during those months of 2013 where the ‘eligibility to supply’ status could be determined, with and without application of the seasonality adjustment and by calculation method. Using calculation method 1 and the seasonality adjustment in place, an estimated 860 (5.1%) herds were liable for suspension during 2013. If the seasonality adjustment were removed, this number would increase to 974 (5.8% of the national population), which is a 13.2% increase in herds liable for suspension. Using calculation method 2 and the seasonality adjustment in place, an estimated 854 (5.1%) herds were liable for suspension during 2013. If the seasonality adjustment were removed, this number would increase to 964 (5.8% of the national population), a 12.9% increase in herds liable for suspension.

**Table 5:** The maximum level of non-compliance reached by Irish herds during those months of 2013 where the ‘eligibility to supply’ status could be determined, by calculation method and with and without application of the seasonality adjustment

The maximum level of non-compliance, with the seasonality adjustment	The maximum level of non-compliance, without the seasonality adjustment					Total (with seasonal adjustments)
	Always compliant	At least one first warning	At least one second warning	At least one third warning	Liable for suspension	
Calculation method 1						
Always compliant	13426	524	60	0	0	14010
At least one first warning	0	607	101	13	0	721
At least one second warning	0	0	496	95	11	602
At least one third warning	0	0	0	444	103	547
Liable for suspension	0	0	0	0	860	860
Total (without seasonal adjustments)	13426	1131	657	552	974	16740
Calculation method 2						
Always compliant	13616	393	48	0	0	14057
At least one first warning	0	542	87	14	0	643
At least one second warning	0	0	513	84	9	606
At least one third warning	0	0	0	479	101	580
Liable for suspension	0	0	0	0	854	854
Total (without seasonal adjustments)	13616	935	648	577	964	16740

The seasonality formula was applied to, and 3-monthly rolling geometric mean calculated using, either mean monthly SCC values (Calculation method 1) or all individual SCC values (Calculation method 2) in each relevant month. Without the application of the seasonality formula, an estimated additional 114 (13.2%) or 110 (12.9%) herds would have been liable for suspension, using calculation methods 1 and 2, respectively

## Discussion

The impact of removal of the seasonality formula was the primary motivation for this study. The study results indicate that the modelled impact of such removal would be relatively minor, based on available data, regardless of the method used to calculate the 3-month rolling geometric mean. The projected increase in the number of herds liable for suspension represents approximately 0.7% (114/16,740) of the national herd.

In agreement with earlier work [15, 16], there is a strongly seasonal pattern of mean SCC, peaking at the start and end of each year. Further, there is a substantial impact of the seasonality formula for the months applied (see the top and bottom graphs in Figs. 2 and 3). It has been suggested that this pattern is physiological in nature; indeed, this is the rationale for the seasonality formula. However, based on recent work, there is no evidence of a dilution effect in Irish dairy cattle [12]. As an alternative, it is suspected, but not yet proven, that the effect in Ireland is pathological; that is, increases in SCC towards the end of the year are a consequence of persistent infections from earlier in the lactation, and the addition of new infections throughout the lactation.

Application of the seasonality formula is highly influential in terms of mean SCC distribution in the national herd over the year [15]. Nonetheless, as shown here and based on the data available to us, the removal of the seasonality formula would not greatly impact on herd eligibility to supply. The reasons for this discrepancy are likely related both to the timing and duration of high SCC in a herd and to the varying influence of data adjustment and interpretation, as allowed under EU and Irish legislation, at different times throughout the year. The 3-month rolling geometric mean and the 3-month recovery period (that is, a maximum of 3 months under warning, prior to suspension) are each applied throughout the year, but the 3 month rolling geometric mean is ‘reset’ in Ireland following a break-in-supply of at least one calendar month [15]. The seasonality formula is only applied in November–February. Therefore, suspension in December would require high SCC from at least the previous August but likely longer, given the use of the 3 month rolling average and 3 months of warning prior to suspension. In contrast, no herd can be liable for suspension from February through to at least July, even if the geometric mean is over 400,000 cells/mL, if a break-in-supply had been applied for at least the full month of January.

Ongoing application of the seasonality formula is a barrier to timely corrective action, on some farms with poor udder health. In the case of non-compliant milk, Regulation No 854/2004 provides for a 3-month window of opportunity, during which action should be taken to correct the situation. However this action may currently be deferred, relying instead on the seasonality factor as a solution. If the seasonality factor were removed, there would be a need for change in behaviour at both milk purchaser and producer level, to take positive corrective action as early as possible.

The relevant EU legislation is open to interpretation, as noted previously [15], which has the potential to influence the eligibility to supply depending on the methodology applied. Hence the decision to use several methods to calculate the 3-month rolling geometric mean in this study. Our methods differ both in terms of the method used to calculate the 3-month rolling geometric mean and the inclusion, or otherwise, of SCC data without a corresponding recorded milk volume. Calculation methods 1 and 2 are both compliant with EU legislation, with calculation method 2 currently applied by DAFM. The two methods used to calculate the 3-month rolling geometric mean would be equivalent provided the monthly number of individual SCC test results is the same. Using calculation method 2, however, the 3-month rolling geometric mean can change substantially with an increase in the number of individual SCC test results, particularly if they are markedly lower (or higher) than other values. Calculation method 1 is more resistant to such interference. The differences observed in Table 5 (for example, the estimated number of herds liable for suspension using calculation methods 1 and 2) are, in part, a reflection of this effect. The inclusion or otherwise of SCC data without a corresponding recorded milk volume can also have a substantial impact when assigning a herd ‘eligibility to supply’ status. We illustrate this point using calculation method 2 on the 2013 data. If these data are excluded, we estimated that the number of herds liable for suspension would increase from an estimated 854 to 964 (5.8% of herds, 12.9% increase in suspensions) herds (Table 5), if the seasonality formula were removed. If these data were not excluded, the number of herds liable for suspension would be an estimated 733, and would increase to 839 (5.0% of herds, 14.5% increase in suspensions) herds with the removal of the seasonality formula (data not shown).

The results of this study need to be interpreted with caution, for several reasons.

The focus of the current study was quite narrow, effectively from July to December 2013. There are several reasons for this. Under current EU and Irish legislation, herd suspension cannot occur during the first 6 months following a break in supply (the 3-month rolling geometric mean cannot be calculated during months 1–3 and therefore the first warning cannot commence prior to month 4) [15], regardless of SCC during that period. In this study, our calculations were based on an enforced apparent ‘break in supply’ for all herds immediately prior to January 2013. Although the study results are robust during July to December 2013, we anticipate differences in both the frequency of herds liable for suspension and the impact of the seasonality formula (and its removal) in the first compared to the second half of each year. In a sizeable percentage of Irish herds, SCC values are highest during the winter months [16]. In these herds, the observed SCC rise is less likely to result in herds liable for suspension in early winter (November–December) compared to late winter and early spring (January–April), specifically because of the time defined under legislation between the start of a sustained period of SCC increase and the month of eventual suspension. Similarly, the seasonality formula is likely less influential in early winter (November–December) compared to late winter and early spring (January–April). The estimated number of herds liable for suspension during 2013 (860 using calculation method 1, 854 using calculation method 2; each in the presence of the seasonality formula) is undoubtedly an underestimate, as it takes no account of herds liable for suspension during January to June 2013. The percentage change in herds liable for suspension with the removal of the seasonality formula (13.2% using calculation method 1, 12.9% using calculation method 2) would also underestimate the true percentage increase if the above hypothesis were true.

For a sizeable percentage of the 2013 bulk tank SCC data, the national herd identifier was not available (some cooperatives supplied their own randomly generated herd code, which could change year on year). In the preliminary analysis, we investigated the possibility of using a subset of the data (that is, data for which a national herd identifier was available along with two consecutive years of data) instead of the complete dataset. However, comparison of usable and unusable data at a monthly level indicated that there was a significant difference in every month for monthly volume records, with a similar result for SCC records (Table 4). In the main analysis, which was based on the full 2013 dataset, our investigation was constrained because the ‘eligibility to supply’ status could not be calculated for any herds during January to April 2013, and for a number of herds subsequently (Fig. 4 and Additional file 2: Figure S1). This is because a rolling geometric mean can only be calculated following an initial ‘burn-in period’ (that is, once 3 months of data were available). A national herd identifier will be needed if data are to be linked across years. It would also have allowed us to identify and account for herds that supply to more than one cooperative during or across years. In the current analysis, which considers only a single year of supply, dual supply could not be accounted for. It is uncertain whether the results from 2013 can be generalised to later years.

## Conclusions

It is estimated that the number of herds liable for suspension would increase by 12.9% (using calculation method 2), from 854 to 964. Removal of the seasonality factor is likely to have a positive impact on udder health, prompting corrective action earlier rather than relying on data adjustment as a short-term solution. This conclusion is based on available data, and needs to be interpreted with caution. The focus of the current study was quite narrow, effectively from July to December 2013, and therefore the results are an underestimation of the total number of herds liable for suspension during 2013. The results from this study should assist with national policy decision-making with respect to SCC data adjustment and interpretation, as outlined in EU legislation, when determining herd eligibility to supply raw milk for processing of dairy products.

## Additional files

Additional file 1: Appendix 1. Pseudocode detailing the two methods for calculating the rolling geometric mean herd SCC and monthly herd compliance. (PDF 83 kb)
Additional file 2: Figure S1. The estimated percentage of Irish herds eligible to supply raw milk for processing of dairy products, by month, using calculation method 1. The seasonality formula was either applied (top) or not (bottom). (PDF 7 kb)
Additional file 3: Figure S2. The relative monthly change in the percentage of national volume under warning and liable for suspension following removal of the seasonality adjustment, using calculation method 1. (PDF 5 kb)
