# Supplementary material for: The impact of removal of the seasonality formula on the eligibility of Irish herds to supply raw milk for processing of dairy products
Source: Ir Vet J. 2017 Feb 23;70:9. doi: 10.1186/s13620-017-0083-z (PMC5322608; doi:10.1186/s13620-017-0083-z)
Supplement: Additional file 1: Appendix 1. — Pseudocode detailing the two methods for calculating the rolling geometric mean herd SCC and monthly herd compliance. (PDF 83 kb) [file 13620_2017_83_MOESM1_ESM.pdf]

Additional file 1

a. Calculation method 1:

Calculation of 3-month rolling average

```
calculate_rolling_mean {
        prev_means = monthly geometric mean from this month and previous two
        consecutive months

        if (length(prev_recordings) < 3) { # early in the data or after a gap in supply
                rolling_mean = NA
        } else {
                rolling_mean = geometric_mean(prev_means)
         }
}
```

b. Calculation method 2:

```
calculate_rolling_mean{
        prev_recordings = all SCC results (taken at the same time as a volume recording)
        from this month and previous two consecutive months
        if (precordings does not include results from 3 consecutive months) {
                rolling_mean = NA
        } else {
                rolling_mean = geometric_mean(prev_recordings)
        }
}
```

Calculation of herd eligibility to supply (applicable to both methods of rolling mean calculation):

```
calculate_supplier_status {
         prev_means = rolling geometric mean from previous four months # = {rolling mean
        from current_month -4, -3, -2, -1}

        if (length(prev_means) < 4) { # Any breaks occurring in the monthly rolling means
                status = "incalculable"
```

```
        } else if (most recent month in prev_means <= 400000) {
                status = "compliant"
        } else if (any earlier month <= 400000) {
                status = "warning"
        } else { # All previous means from recent months are too high
                status = "liable for suspension"
        }
}
```
