# Supplementary figures and images for: The impact of removal of the seasonality formula on the eligibility of Irish herds to supply raw milk for processing of dairy products
Source: Ir Vet J. 2017 Feb 23;70:9. doi: 10.1186/s13620-017-0083-z (PMC5322608; doi:10.1186/s13620-017-0083-z)

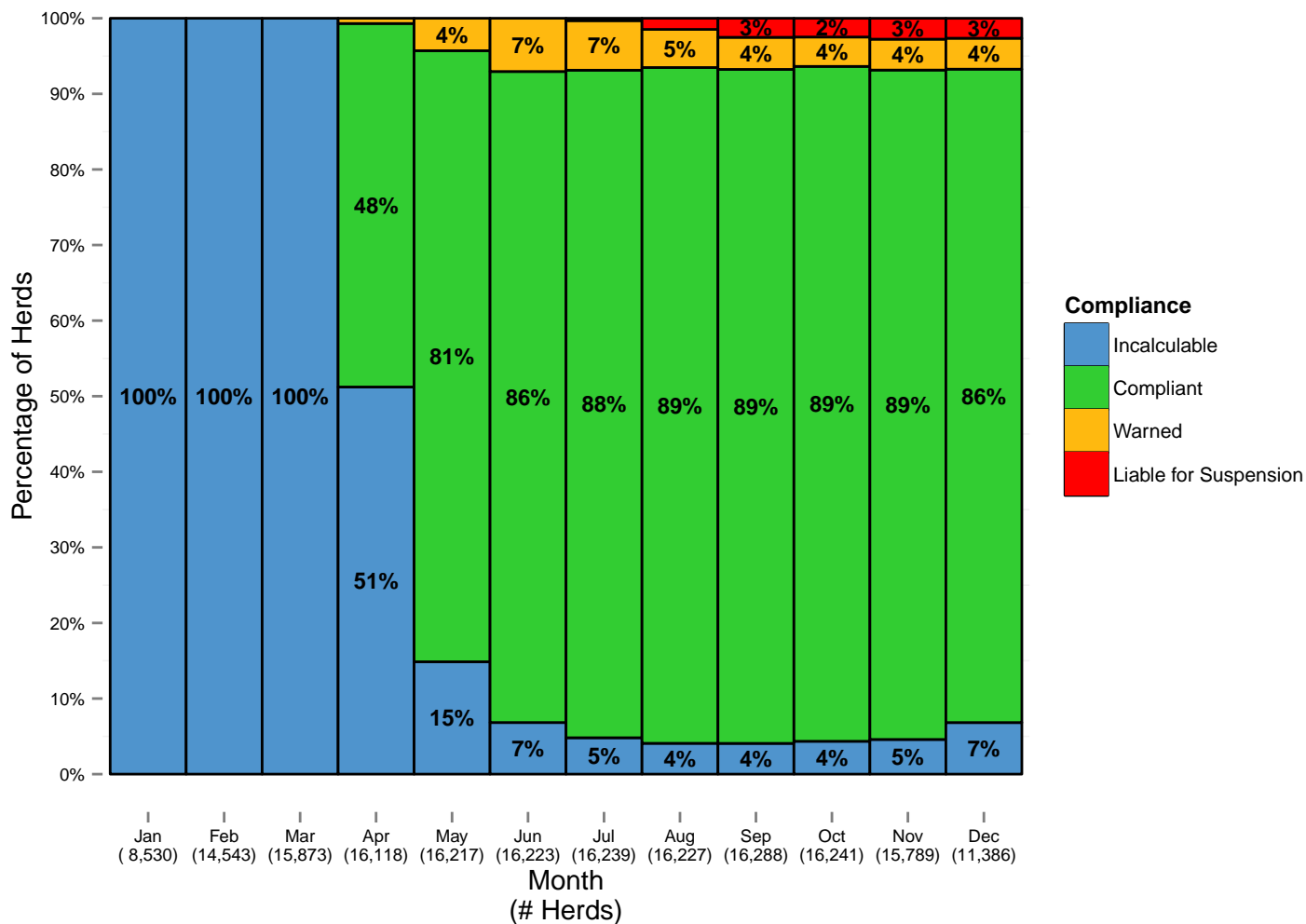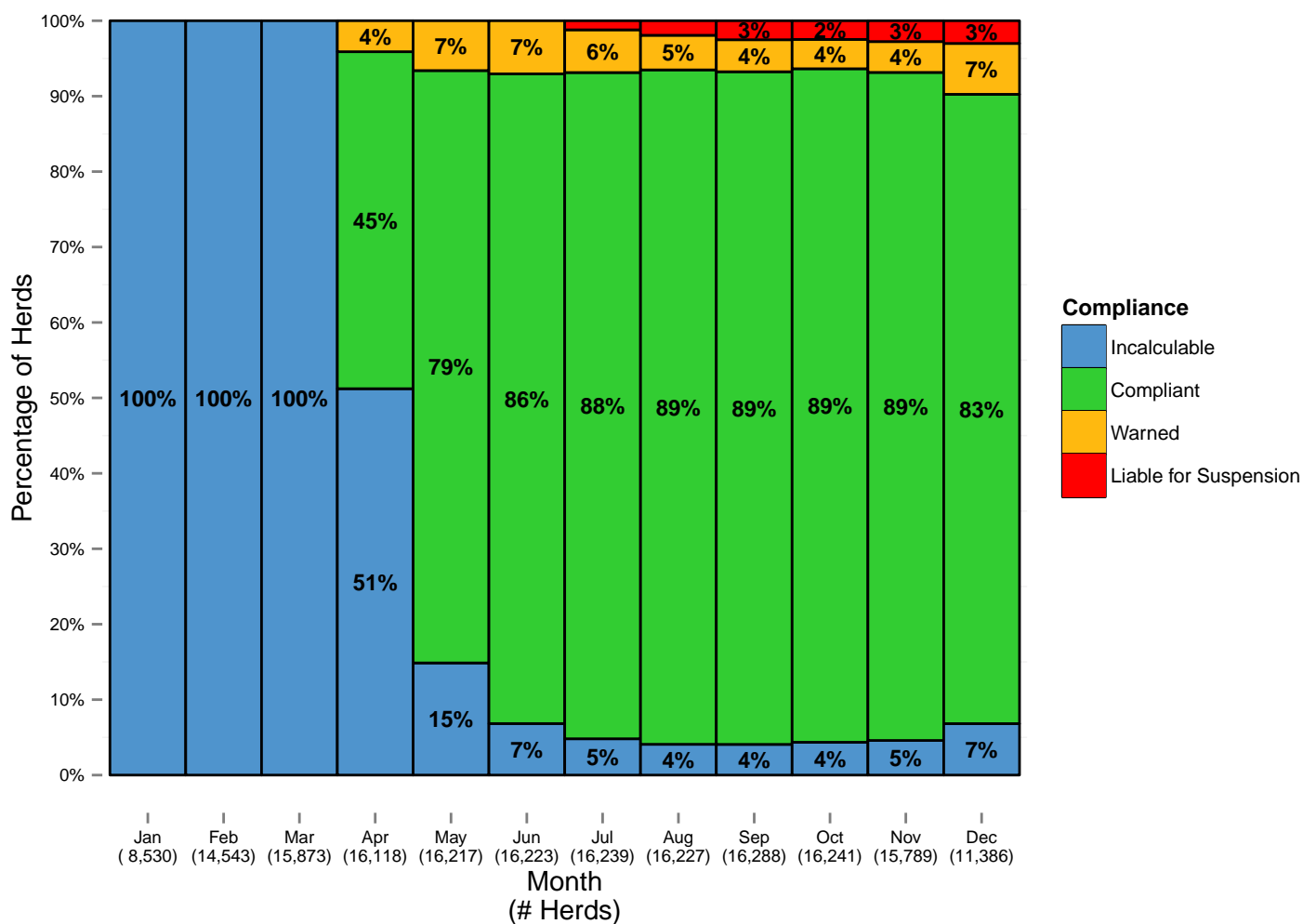

Supplement: Additional file 2: Figure S1. — The estimated percentage of Irish herds eligible to supply raw milk for processing of dairy products, by month, using calculation method 1. The seasonality formula was either applied (top) or not (bottom). (PDF 7 kb) [file 13620_2017_83_MOESM2_ESM.pdf]

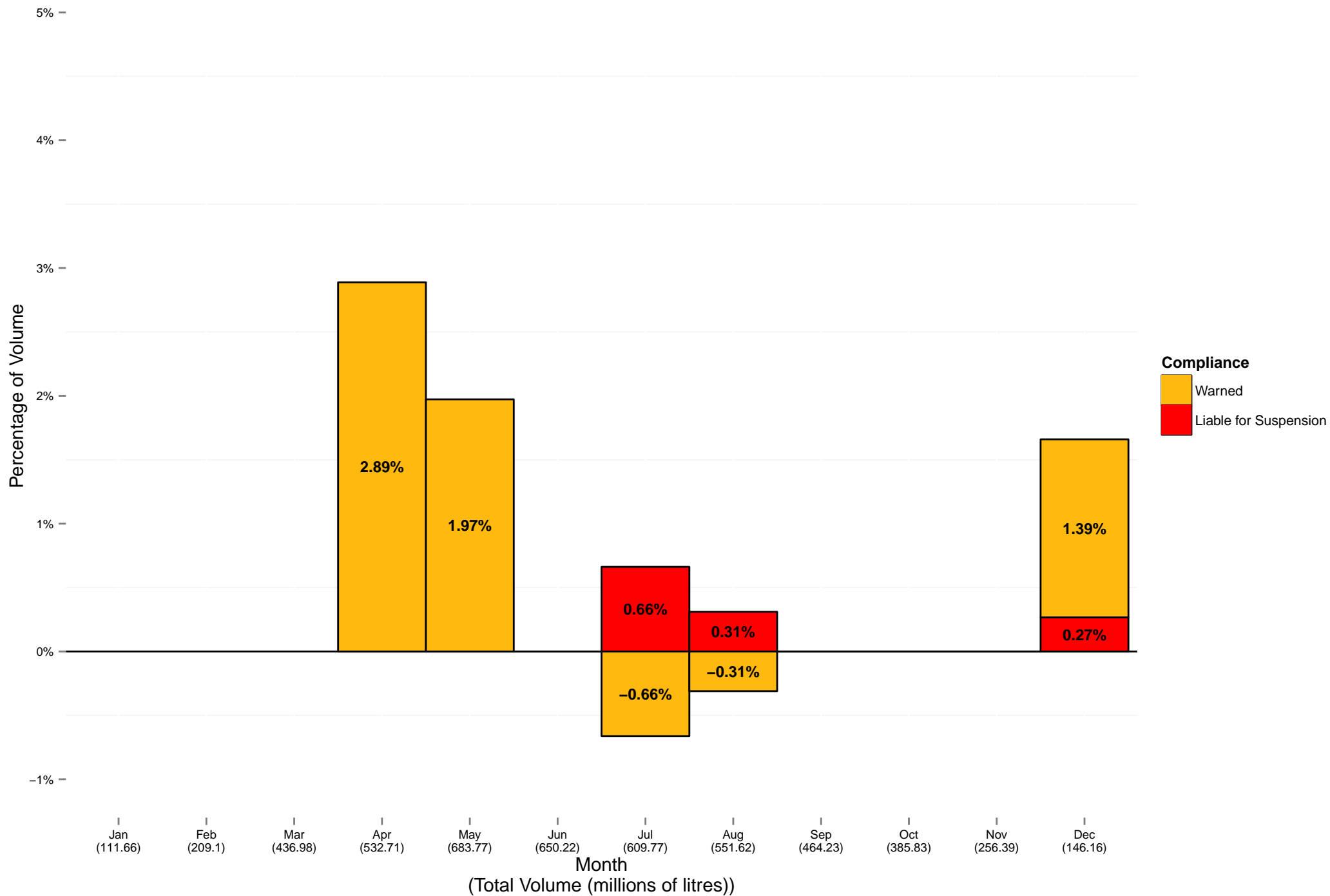

Supplement: Additional file 3: Figure S2. — The relative monthly change in the percentage of national volume under warning and liable for suspension following removal of the seasonality adjustment, using calculation method 1. (PDF 5 kb) [file 13620_2017_83_MOESM3_ESM.pdf]
